# Comparison of serum glycosylated hemoglobin levels in patients with diabetic cystoid macular edema with and without serous macular detachment

**DOI:** 10.4103/0301-4738.67044

**Published:** 2010

**Authors:** Burak Turgut, Fatih Cem Gul, Nevin Ilhan, Tamer Demir, Ulku Celiker

**Affiliations:** Department of Ophthalmology, Firat University School of Medicine, Elazig, Turkey; 1 Department of Biochemistry, Firat University School of Medicine, Elazig, Turkey

**Keywords:** Diabetic cystoid macula edema, HbA1c, retinal pigment epithelium dysfunction, serous macular detachment

## Abstract

**Aim:**

A clinical comparative trial was conducted to compare the levels of glycosylated hemoglobin (HbA1c) in patients with diabetic cystoid macular edema (CME) with and without serous macula detachment (SMD).

**Materials and Methods:**

Thirty patients (group 1) with diabetic CME in both eyes, but without SMD, and 30 patients (group 2) with diabetic CME and SMD in both eyes documented by optical coherence tomography (OCT) and fundus fluorescein angiography (FFA), were included in the study. In addition to the measurement of central macular thickness by OCT and visual acuity (VA) (as logMAR) using the the early treatment diabetic retinopathy study (ETDRS) chart, the concentrations of HbA1c were measured by high performance liquid chromatography (HPLC). Statistical analysis was done by independent samples *t* test.

**Results:**

The mean logMAR VA was 0.8 ± 0.22 (1.0–0.5) in group 1and 0.7 ± 0.16 (1.0–0.6) in group 2. The mean central macular thickness, as determined by OCT, was 468.70 ± 70.44 μm (344–602 μm) in group 1 and 477.80 ± 73.34 μm (354–612 μm) in group 2. The difference between the groups was not statistically significant (*P* = 0.626). The mean HbA1c levels were 8.16 ± 0.99% in group 1 and 10.05 ± 1.66% in group 2. The difference between the groups was statistically significant (*P* < 0.001).

**Conclusions:**

The presence of SMD and high HbA1c levels in the patients with diabetic CME may be indirectly suggestive of retinal pigment epithelium dysfunction.

Macular edema (ME) in diabetic patients is the main reason for loss of vision, and this may be caused by breakdown of the inner and/or the outer blood retinal barrier (BRB). Retinal pigment epithelium (RPE) forms the outer BRB, and it contributes to the normal fluid dynamics in the subretinal space through the intercellular structure. RPE dysfunction is thought to affect the formation and continuity of the ME.[1–3]

Using optical coherence tomography (OCT), Otani *et al*. have described three patterns of diabetic ME: sponge-like swelling, cystoid macular edema (CME) and serous macular detachment (SMD).[4] Because it may hide beneath CME during fundus fluorescein angiography (FFA), SMD associated with CME can only be diagnosed using OCT.[5]

SMD may occur in conditions where retinal vascular leakage or RPE dysfunction is seen, for example, in diabetic ME, branch retinal vein occlusion (BRVO), central retinal vein occlusion (CRVO), hypotonous maculopathy, retinal vasculitis and retinal macro-aneurysm. SMD associated with ME has been demonstrated in 15–46% of eyes with diabetic ME, 38–71% of eyes with BRVO, and 82% of eyes with CRVO.[6–11] Although the exact mechanism of development of SMD is not known, it is probably due to excessive fluid flow from the abnormal retinal vessels, which overwhelms the RPE pump leading to serous retinal detachment.[6,12,13]

The presence of SMD in retinal vascular diseases such as diabetes may affect the treatment results for ME associated with retinal vascular leakage and it may limit the ability to perform effective macular laser treatment. Ohashi *et al* reported that in ME associated BRVO, the presence of SMD is a negative prognostic factor for resolution of ME and for visual acuity (VA) after grid macular laser treatment.[14]

High glycosylated hemoglobin (HbA1c) level is a well-known risk factor for diabetic ME. In addition, the Diabetes Control and Complications Trial (DCCT) demonstrated that intensive treatment to maintain blood glucose levels at a normal range reduced the risk of clinically significant ME at the rate of 23%.[15]

In light of these reports, in the present study, we focused on why SMD is seen in only some diabetic CME patients. Thus, it was considered that hyperglycemia may promote the development of SMD, and that a possible relationship may exist between HbA1c and SMD.

The aim of this study was to compare the levels of HbA1c in the patients with diabetic CME with and without SMD.

## Materials and Methods

The study population consisted of 30 patients with diabetic CME in both eyes but without SMD (group 1) and 30 patients with diabetic CME with SMD in both the eyes (group 2).

Inclusion criteria for this study were as follows:

1. the presence of CME detected by FFA and OCT in both eyes and
2. the presence of CME and SMD documented by OCT in both eyes.

Patients with epiretinal membrane or vitreo-macular traction documented by OCT, and media opacities such as corneal opacity, lens opacity, vitreous and pre-retinal hemorrhage, uveitis, proliferative diabetic retinopathy and patients with history of previous intraocular surgery, macular laser photocoagulation and intravitreal injection were excluded from the study. Patients with uncontrollable systemic hypertension, any renal dysfunction, anemia, pregnancy and psychiatric illness were also excluded from the study.

The patients underwent complete ophthalmic examination, including best-corrected VA measurement (logMAR) using the early treatment diabetic retinopathy study (ETDRS) chart, slitlamp biomicroscopy with a +90 diopter noncontact lens and a Goldmann three mirror contact lens, FFA and OCT. OCT and FFA examinations and collection of venous blood samples were performed on the same day. FFA was performed with a retinal camera (Topcon 50IX, Itabashiku, Tokyo, Japan). OCT examinations were performed using a spectral OCT (OCT/SLO, OTI Inc., Toronto, Canada). During OCT examination, the maculae were scanned on six radial sections including the horizontal, vertical and oblique planes through the center of the fovea. The macular thickness was measured automatically using the topography software built into the OCT device.

**Figure 1:**
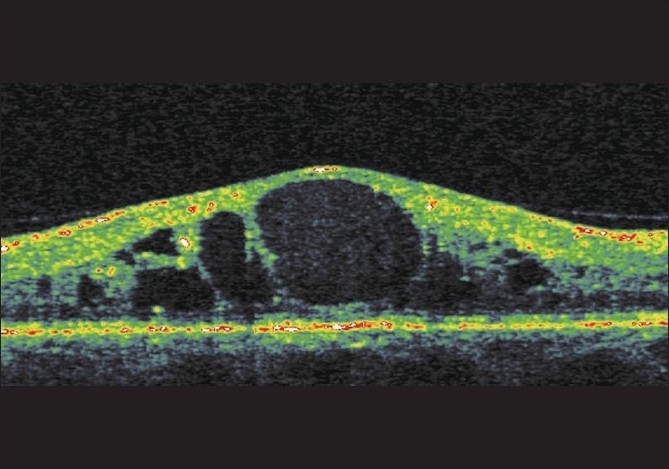
CME in a patient with diabetic retinopathy

Fluorescein angiographic CME was thought to be present if typical oval or petaloid hyperfluorescent cystoid spaces radiating from the fovea were evident during FFA. OCT examination was thought to show CME if there were hyporeflective intraretinal cavities separated by septa and radiating from the center of the macula in cross-sectional scans. Angiography could not detect SMD in any case [Fig. 1].

**Figure 2:**
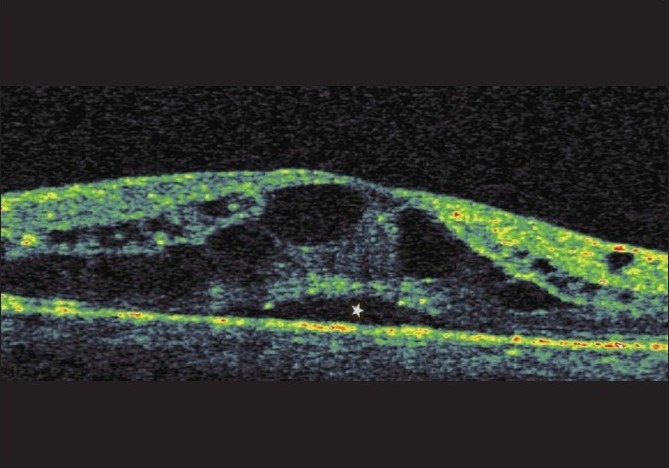
Association of the SMD (star) with CME in a patient with diabetic retinopathy

SMD was thought to be present if the posterior surface of the retina was elevated over a nonreflective black cavity and under the CME, with minimal shadowing of the underlying tissues [Fig. 2].

The OCT, FFA, sample collection and biochemical procedures were performed following the tenets of the Declaration of Helsinki. The study was designed as an institutional, comparative clinical trial and was approved by the institutional ethics committee. Informed consents were obtained from the patients.

The HbA1c measurements were taken with a high performance liquid chromatography (HPLC) method using an HbA1c kit (OSR 6192 kit) and an Olympus AU 2700 (Olympus Corp., Japan) autoanalyzer. The normal HbA1c levels ranged from 4.0 to 6.2% according to the manufacturer’s instructions.

Statistical analysis was performed using the Statistical Package for the Social Sciences, version 11.0 (SPSS, Chicago, IL, USA). The independent samples t test was used to compare numeric variables. Results were presented as means ± standard deviations. P values less than 0.05 were considered significant.

## Results

A total of 30 patients with diabetic CME in both eyes and 30 patients with SMD associated with diabetic CME in both eyes were evaluated. All subjects were phakic. The subjects included 12 men and 18 women in group 1 and 10 men and 20 women in group 2. The mean duration of diabetes was 18.6 years in group 1 and 20.2 years in group 2 with no statistical difference (*P* = 0.156). The mean age was 62.9 ± 6.95 years (48–78) in group 1 and 63.53 ± 6.04 years (52–73) in group 2. The difference between the groups was not statistically significant (*P* = 0.708). In group 1, five patients (16.67%) had diabetes mellitus (DM) type 1 and 25 patients (83.3%) had DM type 2, whereas in group 2, three patients (10%) had DM type 1 and 27 patients (90%) had DM type 2. The difference between the groups was also not statistically significant (*P* = 0.706).

The mean logMAR VA was 0.8 ± 0.22 (1.0–0.5) in group 1 and 0.7 ± 0.16 (1.0–0.6) in group 2.

The mean central macular thickness, as determined by OCT, was 468.70 ± 70.44 μm (344-602 μm) in group 1 and 477.80 ± 73.34 μm (354-612 μm) in group 2. The difference between the groups was not statistically significant (*P* = 0.626). The mean HbA1c levels were 8.16 ± 0.99% (5.7-9.90%) in group 1 and 10.05 ± 1.66% (7.50-15.30%) in group 2 [Table 1]. The difference between the groups was statistically significant (*P* = 0.001).

**Table 1:** Mean central macular thickness, mean HbA1c levels, mean VAs (logMAR) and mean age of the groups

Group	Mean age (years)	Mean central macular thickness (μm)	Mean VA (logMAR)	Mean HbA1c level (%)
Group 1	62.9 ± 6.95	468.70 ± 70.44	0.8 ± 0.22	8.16 ± 0.99
(*n* = 30)			
Group 2	63.53 ± 6.04	477.80 ± 73.34	0.7 ± 0.16	10.05 ± 1.66*
(*n* = 30)			

* indicates statistical significance (*P* < 0.05).

## Discussion

The pathogenesis of SMD associated with diabetic macular edema (DME) is linked not only to an abnormality of the draining vascular system but also to an impairment in the function of the RPE.[6] It is suggested that the pathology of these two entities might share a common pathogenic mechanism.[5–8]

The breakdown of the RPE pump or the disruption of the tight junctions between adjacent RPE cells results in intraretinal edema and SMD.[16–18] The presence of SMD in patients with CME is also an important finding in terms of showing defective RPE cells.

Elevated HbA1c is a well-known risk factor for diabetic ME, and prolonged hyperglycemia is known to significantly increase the rate of ME, while reduction of HbA1c levels with tight glycemic control decreases the rates of clinically significant ME and other microvascular complications.[15,19,20]

In the present study, we observed that the levels of HbA1c were higher in patients with SMD associated with CME. This finding might be considered to indicate that elevated HbA1c might cause breakdown not only in the inner BRB, but also in the outer BRB. The effects of hyperglycemia on RPE might occur via a variety of pathogenic mechanisms. In prolonged hyperglycemia, increasing intracellular levels of glucose, the directing of glucose to the polyol pathway and sorbitol accumulation within cells leads to the production of advanced glycation end products, oxidative stress, osmotic stress and protein kinase C activation. These effects might cause dysfunction of the RPE pump or the breakdown of the outer BRB.[2,3] These conditions might be attributed as causes of the development of SMD in some diabetic patients.[21–28]

In the present study, SMD was not detected in all diabetic CME patients. This might be because of a limitation of the RPE dysfunction or the presence of sufficient residual RPE pump function in the cases in which SMD was not detected.

Although we consider that high HbA1c values might increase the risk of SMD in the patients with diabetic CME, according to the study results, our study has its own limitations. Lipid dysfunction, a factor which could cause diabetic ME, was not evaluated in our study.

We consider that the detection of SMD using OCT in patients with diabetic CME might be an evidence of RPE dysfunction. The presence of SMD, as well as elevated levels of HbA1c in these patients, might be important predictors for the success of the treatment interventions of CME and overall CME prognosis. In this regard, Shukla *et al*. reported that SMD might be a predictor for the resolution of diabetic ME following the injection of intravitreal triamcinolone acetate.[29] Additionally, Ohashi *et al*. observed that the presence of SMD is a negative prognostic indicator for resolution of ME and for VA after grid macular laser treatment in patients with ME secondary to BRVO.[14]

In conclusion, patients with high HbA1c levels might be at a greater risk for SMD. In other words, the diagnosis of SMD in patients with diabetic CME by OCT might suggest that these patients have poorer metabolic control than those who have only CME. Because the presence of SMD might significantly limit the ability to give effective treatments such as macular laser intervention, prolonged hyperglycemia should be brought under control and these treatments for diabetic ME should be postponed until patients become normoglycemic. Histopathologic studies are needed to identify the exact pathogenic mechanism and causes of the RPE dysfunction that lead to the formation of SMD in patients with diabetic CME.
